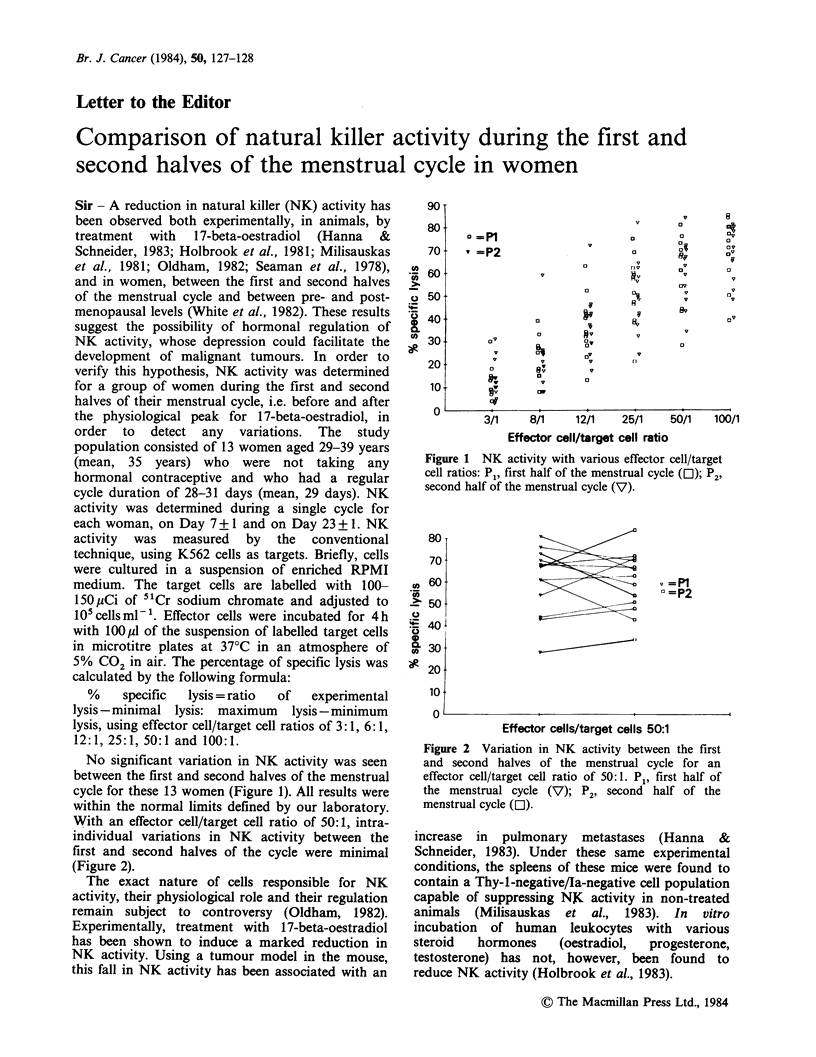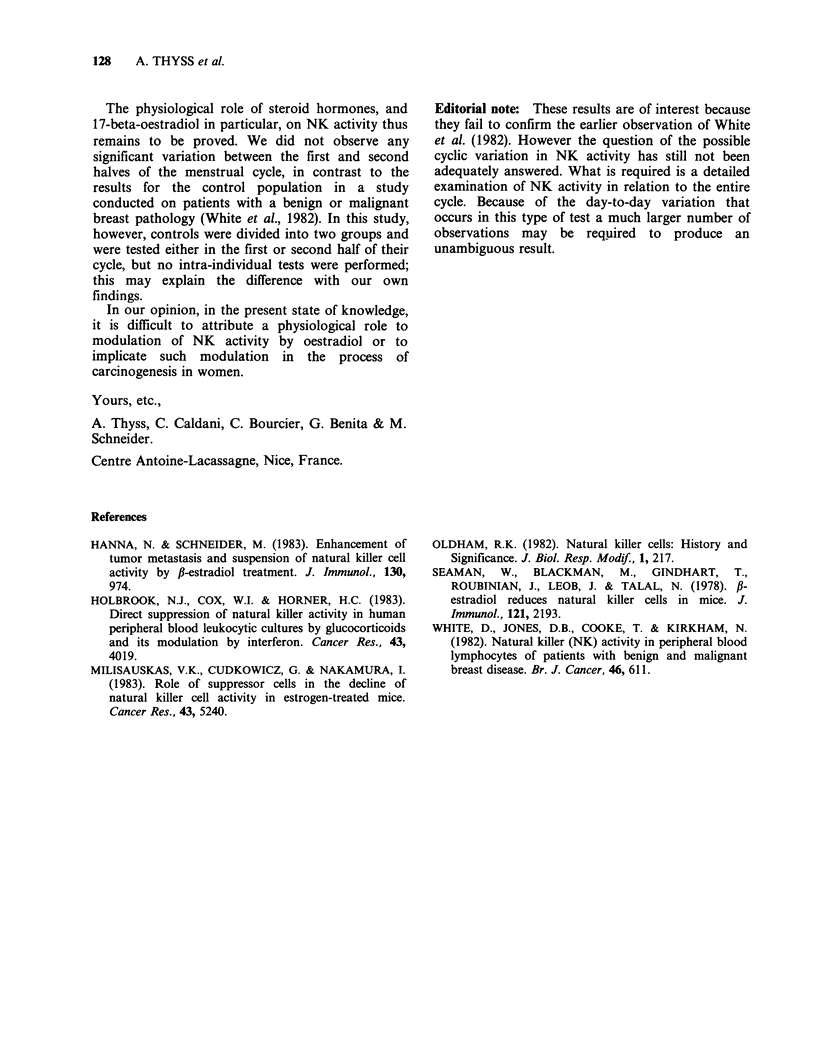# Comparison of natural killer activity during the first and second halves of the menstrual cycle in women.

**DOI:** 10.1038/bjc.1984.149

**Published:** 1984-07

**Authors:** A. Thyss, C. Caldani, C. Bourcier, G. Benita, M. Schneider


*Br. J. Cancer* (1984), **50**, 127–128

### Letter to the Editor

### Comparison of natural killer activity during the first and second halves of the menstrual cycle in women

**Sir** – A reduction in natural killer (NK) activity has been observed both experimentally, in animals, by treatment with 17-beta-oestradiol (Hanna & Schneider, 1983; Holbrook *et al.*, 1981; Milisauskas *et al.*, 1981; Oldham, 1982; Seaman *et al.*, 1978), and in women, between the first and second halves of the menstrual cycle and between pre- and post-menopausal levels (White *et al.*, 1982). These results suggest the possibility of hormonal regulation of NK activity, whose depression could facilitate the development of malignant tumours. In order to verify this hypothesis, NK activity was determined for a group of women during the first and second halves of their menstrual cycle, i.e. before and after the physiological peak for 17-beta-oestradiol, in order to detect any variations. The study population consisted of 13 women aged 29–39 years (mean, 35 years) who were not taking any hormonal contraceptive and who had a regular cycle duration of 28–31 days (mean, 29 days). NK activity was determined during a single cycle for each woman, on Day 7$\pm$1 and on Day 23$\pm$1. NK activity was measured by the conventional technique, using K562 cells as targets. Briefly, cells were cultured in a suspension of enriched RPMI medium. The target cells are labelled with 100–150$\mu$Ci of $^{51}$Cr sodium chromate and adjusted to $10^5$ cells ml$^{-1}$. Effector cells were incubated for 4h with 100$\mu$l of the suspension of labelled target cells in microtitre plates at 37°C in an atmosphere of 5% $CO_2$ in air. The percentage of specific lysis was calculated by the following formula:

% specific lysis = ratio of experimental lysis – minimal lysis: maximum lysis – minimum lysis, using effector cell/target cell ratios of 3:1, 6:1, 12:1, 25:1, 50:1 and 100:1.

No significant variation in NK activity was seen between the first and second halves of the menstrual cycle for these 13 women (Figure 1). All results were within the normal limits defined by our laboratory. With an effector cell/target cell ratio of 50:1, intra-individual variations in NK activity between the first and second halves of the cycle were minimal (Figure 2).

The exact nature of cells responsible for NK activity, their physiological role and their regulation remain subject to controversy (Oldham, 1982). Experimentally, treatment with 17-beta-oestradiol has been shown to induce a marked reduction in NK activity. Using a tumour model in the mouse, this fall in NK activity has been associated with an

**Figure 1** NK activity with various effector cell/target cell ratios: $P_1$, first half of the menstrual cycle (□); $P_2$, second half of the menstrual cycle (▽).

**Figure 2** Variation in NK activity between the first and second halves of the menstrual cycle for an effector cell/target cell ratio of 50:1. $P_1$, first half of the menstrual cycle (▽); $P_2$, second half of the menstrual cycle (□).

increase in pulmonary metastases (Hanna & Schneider, 1983). Under these same experimental conditions, the spleens of these mice were found to contain a Thy-1-negative/Ia-negative cell population capable of suppressing NK activity in non-treated animals (Milisauskas *et al.*, 1983). *In vitro* incubation of human leukocytes with various steroid hormones (oestradiol, progesterone, testosterone) has not, however, been found to reduce NK activity (Holbrook *et al.*, 1983).

© The Macmillan Press Ltd., 1984

The physiological role of steroid hormones, and 17-beta-oestradiol in particular, on NK activity thus remains to be proved. We did not observe any significant variation between the first and second halves of the menstrual cycle, in contrast to the results for the control population in a study conducted on patients with a benign or malignant breast pathology (White *et al.*, 1982). In this study, however, controls were divided into two groups and were tested either in the first or second half of their cycle, but no intra-individual tests were performed; this may explain the difference with our own findings.

In our opinion, in the present state of knowledge, it is difficult to attribute a physiological role to modulation of NK activity by oestradiol or to implicate such modulation in the process of carcinogenesis in women.

Yours, etc.,

A. Thyss, C. Caldani, C. Bourcier, G. Benita & M. Schneider.

Centre Antoine-Lacassagne, Nice, France.

**Editorial note:** These results are of interest because they fail to confirm the earlier observation of White *et al.* (1982). However the question of the possible cyclic variation in NK activity has still not been adequately answered. What is required is a detailed examination of NK activity in relation to the entire cycle. Because of the day-to-day variation that occurs in this type of test a much larger number of observations may be required to produce an unambiguous result.

**References**

HANNA, N. & SCHNEIDER, M. (1983). Enhancement of tumor metastasis and suspension of natural killer cell activity by β-estradiol treatment. *J. Immunol.*, **130,** 974.

HOLBROOK, N.J., COX, W.I. & HORNER, H.C. (1983). Direct suppression of natural killer activity in human peripheral blood leukocytic cultures by glucocorticoids and its modulation by interferon. *Cancer Res.*, **43,** 4019.

MILISAUSKAS, V.K., CUDKOWICZ, G. & NAKAMURA, I. (1983). Role of suppressor cells in the decline of natural killer cell activity in estrogen-treated mice. *Cancer Res.*, **43,** 5240.

OLDHAM, R.K. (1982). Natural killer cells: History and Significance. *J. Biol. Resp. Modif.*, **1,** 217.

SEAMAN, W., BLACKMAN, M., GINDHART, T., ROUBINIAN, J., LEOB, J. & TALAL, N. (1978). β-estradiol reduces natural killer cells in mice. *J. Immunol.*, **121,** 2193.

WHITE, D., JONES, D.B., COOKE, T. & KIRKHAM, N. (1982). Natural killer (NK) activity in peripheral blood lymphocytes of patients with benign and malignant breast disease. *Br. J. Cancer*, **46,** 611.